# Impact of Environmental Regulation on Scientific and Technological Competitiveness of Resource-Based Cities in China—Based on Panel Data of 33 Resource-Based Cities

**DOI:** 10.3390/ijerph17249187

**Published:** 2020-12-09

**Authors:** Xiaoning Zhang, Mei Qu

**Affiliations:** College of Economics and Management, Northwest A&F University, Yangling 712100, China; xiaoningzhang@nwafu.edu.cn

**Keywords:** resource-based city, environmental regulation, scientific and technological competitiveness

## Abstract

After the economy enters a “new normal” era in China, resource-based cities are under pressure in terms of transformation, upgrading and sustainable development. This paper uses the panel data of 33 resource-based cities from 2008 to 2018 to empirically analyze the impact of environmental regulation and innovation compensation on scientific and technological competitiveness. The results show that there is a positive U-shaped relation between environmental regulation and scientific and technological competitiveness. This means that when environmental regulations exceed a certain level, continuing to increase regulations will significantly enhance technological competitiveness, but most samples are still on the left side of the turning point. At the same time, the labor productivity and fiscal capacity of non-agricultural industries in the region may have a strong regulatory effect. In a region with higher labor productivity in non-agricultural industries or stronger local fiscal capacity, environmental regulation is more likely to reflect the attribute of “innovation compensation” and advance scientific and technological competitiveness. At this stage, we should optimize the trans-regional compensation mechanism for resource-rich regions, increase investment in pollution management and ecological protection and impose stricter admission standards on industrial projects. Besides, skilled laborers should be cultivated and innovation and entrepreneurship be supported to realize the green and sustainable development of resource-based cities in the new era.

## 1. Introduction

With a background of strengthening the development of a more ecological civilization and expanding employment, can environmental regulation achieve the multiple goals of environmental protection, green employment and high-quality development, simultaneously? This has become a major issue that needs to be resolved urgently. Since reform and opening up, China’s economy has continued to grow rapidly for more than 40 years, but over these years extensive development has also brought about many problems such as structural imbalance, serious waste of resources, and decline in environmental quality (Ebensteina et al., 2015) [1]. In particular, smog has concentrated over a large area, seriously damaging economic efficiency, public health and government image. Reshaping the dynamic mechanism of economic growth and realizing the transition from rapid economic growth to high-quality development has become a major issue that needs to be resolved (Chen et al., 2018) [2]. In recent years, the academic community has carried out beneficial explorations on the motivation mechanism for high-quality development, mainly from the perspectives of economic growth, technological innovation, and industrial upgrading. However, compared with these direct incentives, environmental regulations are an important part of local government governance, but their impact on high-quality development has not received enough attention (Acemoglu et al., 2012; Yang et al., 2017, Aghion P et al., 2015) [3,4,5,6]. Although the starting point of environmental regulation is to control pollution, the traditional view is that this will increase the production cost of enterprises, which will inevitably damage the quality of economic development.

The large number of resource-based cities in China is an important carrier for the initial development of modern industry in China and a guarantee of the current safeguards for energy and resources. According to the National Sustainable Development Plan for Resource-based Cities (2013–2020) [7], there are 262 resource-based cities in China, 79.3% of which are mature or declining cities, and 69 are resource-exhausted cities. Compared with developed countries, China’s resource-based cities have entered the exhausted stage more concentratedly (Li, 2015) [8]. With the depletion of natural resources and the transformation of economic growth mode, resource-based regions have gradually become the “collapsed areas” of China’s economic development. The sustainable development of resource-based cities is an important part of transforming the mode of economic development. This requires strengthening the prevention and control of major pollutants, vigorously promoting energy conservation and emission reduction, and promoting green development of circulation and low carbon. The new normal economy puts resource-based cities under pressure for transformation upgrading and sustainable development, because the sustainable development of resource-based cities is an important part of transforming the mode of economic development. However, most resource-based cities face such challenges as a single industrial structure, lagging production technology, low-level productivity and slow development of alternative industries. It is urgent to promote scientific and technological competitiveness and facilitate construction of a diversified modern industrial system in an all-round way by increasing investment in innovation. If environmental regulation in resource-based cities is able to drive “innovation compensation” and technological progress of enterprises, and to promote environmental quality and transformation and upgrading of industrial structure to sustain economic growth, it will be of great significance for the sustainable development of resource-based cities in the “new normal” economy.

The positive significance of environmental regulation for the sustainable development of economy and society has never been underestimated, but there is controversy about its impact on industrial competitiveness. In particular, in developing regions with relatively weak industrial foundations, excessive environmental responsibilities undertaken by enterprises may curb profit growth and industrial upgrading. In theory, there are two opposite views on the industrial effect of environmental regulation: compliance cost and innovation compensation (Freeman et al., 1972) [9,10,11,12,13]. According to Freeman and Haveman (1972) [9] and Dension (1984) [10], with the principle of “polluter pays”, the increasing burden of cost on pollution control and governance and energy conservation and emission reduction turns out to be “compliance cost”, to the disadvantage of industrial development. Porter and Linde (1995) [11] considered that environmental regulation not only crowded out factor inputs of research and development and production, but also stimulated enterprises to enhance innovation in order to increase factor productivity. This can offset “compliance cost” and help enterprises form a new competitive advantage, that is, “innovation compensation”. Therefore, whether a region can obtain the double bonus of environmental and industrial development lies in whether enterprises can realize innovation compensation and whether environmental regulation can have a positive effect on scientific and technological competitiveness (Gollop et al., 1983; Gray et al., 1988) [12,13], (Jaffe et al., 1997) [14], (Lanoie et al., 2011) [15], (George et al., 2017) [16]. It is important to study the relationship between environmental regulations and regional technological competitiveness in the process of China’s economic transformation from high-speed growth to high-quality development.

The structure of this paper is arranged as follows: the second part will set up a theoretical framework to analyze how resource endowment and the compensation policy for quitting rural residential land will affect migrant workers’ willingness to buy houses in cities; the third section will provide a statistical and descriptive analysis of the collected data; the fourth part intends to build a logistic model to test the influence of individual resource endowment, coupled with the compensation policy for quitting rural residential land, on migrant workers’ willingness to buy houses in cities; the fifth section will make a summary and present some enlightenment for future policy making.

## 2. Literature

Many studies have demonstrated the “innovation compensation” effect of environmental regulation through theoretical or empirical analysis. For example, Mohr (2002) [17] thought that the “compliance cost” effect of environmental regulation is short-term, because in the face of the cost pressure brought by environmental regulation, enterprises would pay more attention to investing in research and development to improve labor productivity and thus maximize profit (Jean et al., 1996) [18]. Zhang et al. (2013) [19], found that in both developed and developing countries, the number of environmental patents climbed with the increase of investment in pollution control, and in turn led to technological progress [20].

Using the manufacturing data of OECD countries, Plouffe and Lanoie (2010) [21] tested the impact of environmental policies on technological innovation. The results showed that the former pushed up the relative prices of environmental factors in the production process, forcing enterprises to develop new types of product that reduced the input of environmental factors and increased production technology. In conclusion, most people hold that although pollution control investment has a certain crowding-out effect on factor input, it will increase investment in technology research and development and “innovation compensation” will thus be obtained from the improvement of science and technology and factor productivity.

Most empirical analysis of China’s data found that the “innovation compensation” effect of environmental regulation has a non-linear characteristic and large regional or industrial heterogeneity. For example, Zhang et al., (2011) [20], based on the data from China’s industrial sector, found a U-shaped relation between the strength of environmental regulation and the production technology of enterprises through empirical study, and this U-shaped relation showed significant regional heterogeneity. Shen and Liu (2012) [22] found a U-shaped relation between the intensity of environmental regulation and technological innovation, and this showed regional heterogeneity with differences in economic development. Jiang et al. (2013) [19] used the manufacturing data of Jiangsu Province to demonstrate the U-shaped relation between the intensity of environmental regulation and technological innovation, and FDI (Foreign Direct Investment), firm size and human capital played a clear regulatory role. Differently, Li et al. (2013) [23] and Han et al., (2013) [24] found that the intensity of environmental regulation and technological innovation are in inverted U-shaped relation, and considered the regulatory role of FDI and firm size is especially significant.

According to the Chinese literature, in order to make environmental regulation play the role of “innovation compensation”, regulation intensity should be kept within a suitable range, with guarantees to certain regions or industries. However, in slowly-developing areas with weak pollution control, investment capacity, a single industrial structure, low economic development and openness, and scarce human capital, it is often difficult to achieve the “innovation compensation” effect of environmental regulation.

As can be seen from the previous literature, there is a generally U-shaped relation between environmental regulation and scientific and technological competitiveness. That is, only when regulatory power reaches a certain level can it play a positive role in the improvement of scientific and technological competitiveness, or it will not be conducive to technological progress. Besides, the level of labor skills and local financial capacity play a regulatory role in the relation between environmental regulation and scientific and technological competitiveness. On the basis of previous research, this paper will take resource-based cities as the research object to test the relationship between the intensity of environmental regulations and regional technological competitiveness. Because there are many resource-based cities in China, the main targets of environmental regulation are these cities.

China’s resource-based cities have long specialized in extraction and primary production and processing of energy and resources, and some regions have not yet formed an endogenous growth mechanism, resulting in a limited local fiscal scale. Besides, the drain of highly skilled labor makes it difficult to effectively accumulate human capital. In order to explore the dominant impact of environmental regulation in resource-based cities on scientific and technological competitiveness as “innovation compensation” or “compliance cost”, this paper will take 33 resource-based cities in China (Appendix A) as an example to study the impact of environmental regulation on the competitiveness of science and technology, and then analyze constraints affecting the technological progress of resource-based cities in China. However, there is a problem that needs to be explained: why only 33 cities from China’s 262 resource-based cities used as samples? The main reason is that, considering the availability and integrity of data, the 2008–2018 China Environment Yearbook only included 33 typical resource-based cities in the statistics for GDP, industrial wastewater, and waste gas emissions and treatment in key cities. Therefore, this paper selects these 33 resource-based cities as the empirical test samples. On the basis of the existing research, this paper empirically analyzes the impact of $LP_{it}$ environmental regulation in resource-based cities on scientific and technological competitiveness. The relevant conclusions will provide policy implications for resource-based cities to speed up the transformation of economic development and achieve green endogenous growth.

## 3. Econometric Models and Variables

### 3.1. Econometric Model Settings

Based on the reality of resource-based cities in China and the conclusion of existing studies on the nonlinear relation between environmental regulation and scientific and technological competitiveness, this paper constructs the following econometric model. It contains linear terms and quadratic terms for environmental regulation intensity and can be used to test whether a non-linear relation exists between environmental regulation and scientific and technological competitiveness.

(1) $$Tech_{it}=\alpha_{i}+\beta_{t}+\gamma_{1}ER_{it}+\gamma_{2}ER_{it}^{2}+\delta C_{it}+\varepsilon_{it}$$

In the Formula (1), *Tech_it_* is the scientific and technological competitiveness in the *t* th year of the *i* region; *ER_it_* is the environmental regulation intensity in the *t* th year of *i* region; *ER_it_*^2^ is the quadratic term of environmental regulation intensity; *C_it_* refers to a series of control variables, including the level of regional economic development, the average size of the business and the type of city. α*_i_* is the fixed effect, *β_t_* time effect, *ε_it_* error term, *γ_1_* and *γ_2_* the coefficients. If the coefficient of *ER_it_^2^* is significant and opposite to the sign of the coefficient of *ER_it_*, the non-linear effect of environmental regulation on technological competitiveness can be verified.

Besides, this paper will introduce level of regional labor skills and local financial capacity as the regulatory variables to observe their roles in the mechanism of environmental regulation affecting scientific and technological competitiveness.

(2) $$Tech_{it}=\alpha_{i}+\beta_{t}+\gamma_{1}ER_{it}+\gamma_{2}lnLP_{it}+\gamma_{3}\left(ER_{it}\times lnLP_{it}\right)+\delta C_{it}+\varepsilon$$

(3) $$Tech_{it}=\alpha_{i}+\beta_{t}+\gamma_{1}ER_{it}+\gamma_{2}FA_{it}+\gamma_{3}\left(ER_{it}\times FA_{it}\right)+\delta C_{it}+\varepsilon$$

In the Formulas (2) and (3), *lnLP_it_* refers to the level of labor skills in the *t* th year of the *i* region, and *FA_it_* refers to the fiscal capacity in the *t* th year of the *i* region. *ER × lnLP_it_* and *ER_it_ × FA_it_* are the interaction terms of environmental regulation with the level of regional labor skills and the local fiscal capacity respectively, and their estimated coefficients reflect the magnitude of regulatory effect.

According to Jaccard and Turris (1990) [25], the overall goodness of fit of the equation increases with the addition of the interaction term, and the interaction coefficient is significantly positive. This indicates that the larger adjustment variable brings stronger positive explanation of the core explanatory variable to the explained variable.

### 3.2. Variable Selection and Processing

#### 3.2.1. Explained Variable

In some literature, scientific and technological competitiveness is measured by measuring the rate of progress of production technology and establishing production function with technological innovation as output. Besides, investment in R & D is often applied. This paper considers that setting R & D investment as a measure of competitiveness in science and technology ignores the process of input-output conversion. In particular, this paper is based on the data of resource-based cities, so the conversion efficiency of R & D input is even more important. Although the former two methods reflect the competitiveness of science and technology in a more comprehensive way, due to the lack of relevant data on investment of resource-based cities in technological innovation, this paper uses the proportion of the number of employees in scientific research and technical service industries to the total number of employees in each city to measure *Tech_it_*, the scientific and technological competitiveness of resource-based cities.

#### 3.2.2. Core Explanatory Variable

When measuring the intensity of environmental regulations, the following methods are often used in the previous literature: Investment in pollution control. Higher pollution control investment reflects stricter environmental standards. Gray and Shadbegian (1993) [13] and Brunnermeier and Cohen (2003) [26] use operating cost data to measure the intensity of environmental regulations; Jaffe and Palmer (1997) [14] use pollution control investment measures to test the impact of supervision on the “compliance costs” of environmental regulations; Zhang et al. (2011) [20] examined the operating expenses and investment in environmental protection equipment undertaken by enterprises to measure the intensity of environmental regulations. The cost of pollution control and emission reduction accounts for the proportion of total output value or industrial added value. This method is used to examine the intensity of environmental regulations from the perspective of the cost borne by enterprises in pollution control. If the company bears higher costs, this indicates strict environmental standards. For example, Levinson and Taylor (2008) [27] used the US Pollution Abatement Operating Cost as a percentage of manufacturing value, added to measure the intensity of environmental regulations; however, Ederington and Minier (2003) [28] used the US Pollution Abatement Operating Cost as a percentage of the total manufacturing cost to measure the intensity of environmental regulations. This method is widely used and is also reflected in Berman and Bui (2001) [29], Lanoie et al., (2008) [30], Zhang et al., (2011) [20], etc. The intensity of supervision over pollution behavior, whether a company faces environmental supervision from the government or the public, will reflect the intensity of environmental regulations to a certain extent. Gray et al. (1993) [31] once observed that whether a manufacturer receives environmental supervision can reflect the strictness of environmental standards. Brunnermeier and Cohen (2003) [26] used environmental regulatory agencies to inspect and supervise corporate pollutant emissions to measure the intensity of environmental regulations; Zhang et al. (2011) [20] believe that public supervision and reporting of corporate pollutant emissions belong to “public participation environmental regulations”; the more times this occurs, the stronger the environmental regulation, so the number of local environmental letters and visits is used as a measure. (The pollutant treatment rate, under strict environmental regulations, means that, the greater the feasibility of the treatment of pollutants, the lower the pollution discharge. Therefore, the pollutant treatment rate can be used as a measure of the intensity of environmental regulations. For example, Li et al., (2013) [32] used industrial SO_2_ removal rate, industrial wastewater discharge compliance rate, and industrial solid waste comprehensive utilization rate to comprehensively measure the degree of environmental regulation constraints in different industries; similarly, Fu and Li (2010) [33] used the above three measures, and the removal rate of smoke and dust was added to measure the severity of pollution emissions in different industries in a similar manner. Changes in pollution emissions. The positive correlation between pollutant emissions and the intensity of environmental regulations is used in this method, and reflects changes in the intensity of environmental regulations compared with the range of changes in the emissions of pollutants such as waste gas and wastewater (Sancho et al., 2000; Domazlicky and Weber, 2004) [34,35], etc.

In order to accurately reflect the differences in the intensity of industrial pollution management in resource-based cities, the first method was chosen in this paper: to collect the current costs of industrial sewage and waste gas treatment equipment operation based on the availability and homogeneity of data. *ER_it_* refers to core explanatory variable. This is equal to the ratio of the total costs of industrial sewage and waste gas treatment equipment to the industrial output value of enterprises above designated size (the main business revenue over 20 million yuan).

#### 3.2.3. Regulatory Variable

In this paper, *lnLP_it_*_,_ the level of regional labor skills, and *FA_it,_* the local fiscal capacity, are selected as regulatory variables as follows. Jiang et al. (2013) [19] pointed out that there was a significantly positive correlation between human capital accumulation and technological innovation capacity. Technological innovation relies on science and technology practitioners’ skills in learning, assimilation and internalization, so a greater stock of human capital in the region is more conducive to the improvement of technological competitiveness.

Therefore, this paper considers that *lnLP_it_*, the level of regional labor skills, is an important factor influencing the realization of the “innovation compensation” effect of environmental regulation. This is measured by the natural logarithm of the gross output of secondary and tertiary industries divided by the number of employees of secondary and tertiary industries in each city.

Li (2013) [32] argued that, through financial decentralization, the difficult task of balancing local economic development with environmental protection is, in effect, turned over to local governments. Henceforth, local fiscal capabilities are directly connected to the environmental awareness of local governments. In other words, the greater the local fiscal capabilities, the more local governments invest in protecting the environment. This, in turn, makes it more likely for environmental regulation to overcome the inflection point in order to realize innovation compensation. This paper measures the variable *FA_it_* using the ratio of cities’ public fiscal expenditures to local gross production.

#### 3.2.4. Control Variable

As the scientific and technological competitiveness of resource-based cities is subject to their own conditions, four control variables are set in this paper.

- *lnGDP_it_*, the level of regional economic development, has a significantly positive impact on the competitiveness of science and technology. As the level of regional economic development continues to improve, R & D investment will increase, forming a strong impetus to the competitiveness of science and technology (Chen, 2011) [36]. This paper measures this variable based on the natural logarithm of GDP per capita in each city.
- *FDI_it_*, the intensity of foreign investment. The technology spillover effect of foreign direct investment is a strong impetus in enhancing the competitiveness of science and technology in a region. Therefore, greater FDI introduction means stronger scientific and technological competitiveness (Chen et al., 2007; Qun, 2008) [37,38]. This variable is measured by the ratio of the actual amount of used foreign capital in the GDP in each city.
- *Size_it_*, the average size of the business. Abundant capital and strong risk tolerance are the scale advantages of an enterprise, and could support technological innovation, a task with a high risk requiring a large investment. In this paper, we divide the gross value of industrial output above designated size (the main business revenue over 20 million yuan each year) by the number of enterprises and take the natural logarithm to measure this variable.
- *Type_it_*, the type of city. According to Planning on Sustainable Development of Resource-Based Cities (2013–2020) [7], resource-based cities are divided into four types: growing city, mature city, recessionary city and regenerative city. The type of sample city affects the study result in the report. In the model, dummy variables D_1_, D_2_ and D_3_ represent the four types, respectively. If the city is a relevant type, the score is 1, otherwise, 0.

The measurement methods for variables and data sources are shown in Table 1.

### 3.3. Data Source

The data on resource-based cities, including regional GDP, total output of industrial enterprises with annual revenue of 20 million yuan or more from main business operations, the number of industrial enterprises, the number of employees and public financial expenditure, and the amount of foreign direct investment in actual use, are from China City Statistical Yearbook (2009–2019) [39]. The expenses for the operation cost of equipment for curbing industrial sewage and waste gas is from the China Environment Yearbook, covering 33 resource-based cities from 2008 to 2018.

## 4. Empirical Results and Analysis

### 4.1. Test Results for U-shaped Relation

First of all, we observe whether there is a positive U-shaped relation between the industrial pollution regulation of resource-based cities and scientific and technological competitiveness. Table 2 shows the test results of the impact of the linear term and quadratic term on scientific and technological competitiveness. Among these, columns (1) and (2) contain only the linear term of *ER_it_*, and column (3) and (4) contain the linear and quadratic term of *ER_it_*. The Hausman test results of all columns support the random effect model of the panel data. From columns (1) and (2), we can see that the coefficient of *ER_it_* is significantly negative at the level of 10%, no matter whether control variables like *lnGDP_it_* are included. Moreover, compared with column (1), the goodness of fit and the overall significance of column (2) are greatly improved with the addition of control variables, and the absolute value and the significance level of the coefficients are slightly improved. In other words, each percentage point increase in environmental regulation in resource-based cities will significantly reduce the proportion of employees in the technological industry by 0.03 of a percentage point. From column (3) and (4), we can see that, with the addition of the quadratic term of *ER_it_*, the absolute value of the coefficient of *ER_it_* linear term increases and the quadratic term is significantly positive at the level of 5%. This shows that there is a positive U-shaped relation between environmental regulation and scientific and technological competitiveness in resource-based cities. Higher investment in the treatment of industrial sewage and waste gas and greater environmental regulation make it more likely to weaken the inhibitory effect of sewage and waste gas on the development of science and technology. There is a U-shaped inflection point in the process.

According to the performance of the *ER_it_* linear term in column (1) and (2), for most resource-based cities, the investment in environmental regulation has not yet crossed the inflection point, and the efficiency loss due to “compliance cost” has not yet been sufficiently compensated by “innovation compensation”. Thus, it is necessary for most resource-based cities to further implement strict standards of industrial pollutants discharge and increase investment in environmental protection equipment operation.

In addition, the performance of each control variable in Table 2 shows that the impact of *lnGDP_it_*, the economic development level, on scientific and technological competitiveness in the region is significantly negative. This result is inconsistent with that based on the national sample.

This may be related to the economic development characteristics of resource-rich regions. The growth of per capita income in these cities mainly depends on the development of natural resources and the related industrial processing chains, and the “crowding-out effect” of the development of such industries on technology service industries is greater than that of “crowding-in effect”. The coefficients of *FDI_it_*, the intensity of using foreign investment, and *Size_it_*, the average size of enterprises basically meets expectations, and they are significantly positive at the level of 5% or 10%. That is, the development of foreign-invested enterprises and large enterprises is conducive to innovation in science and technology. The dummy variables of resource-based cities show that, compared with growing and regenerative cities, the proportions of employees in the technology service industry in mature and recessionary cities are significantly lower and the regional competitiveness of science and technology is relatively weaker.

### 4.2. The Rest Result of the Adjustment Effect

This test is to further decide the relation between industrial pollution control and scientific and the technological competitiveness of resource-based cities and whether the level of labor skills and the local fiscal capacity regulate this relation. Table 3 shows the result of this test on the regulatory effect of environmental regulation and the scientific and technological competitiveness of resource-based cities taking *lnLP_it_,* the level of labor skills, and Fait, the local fiscal capacity, as the regulatory variable. As shown in the table, based on the Hausman test result, all columns should adopt the regression of the random effect panel data. It can be seen from column (1) and (3) that the addition of *lnLP_it_* and Fait cannot significantly change the sign and the significant level of the *ER_it_* coefficients, and the impact of environmental regulation on scientific and technological competitiveness is still significantly negative. The impact of *lnLP_it_*, the level of labor skills, on scientific and technological competitiveness is significantly positive. This indicates that the technological progress of resource-based cities significantly depends on the labor productivity of secondary and tertiary industries. The coefficient of local fiscal capacity is negative, but even this significance is at the level of 10%, so the null hypothesis still cannot be rejected. That means that local fiscal expenditure has a not significantly negative impact on the competitiveness of science and technology. In column (2) and (4) of Table 3, *ER_it_ × lnLP_it_* and *ER × FA_it_* interaction terms of the core explanatory variable and regulatory variable are added, respectively. The results show that the coefficients of *ER_it_ × lnLP_it_* and *ER × FA_it_*, the interaction terms, are positive, and are significant at the level of 5% and 10%. Meanwhile, compared with column (1), the goodness of fit and overall significance of column (2) improve and the coefficient of determination increases by about one percentage point. According to Turrisi & Jaccard (1990) [25], in the variance of regression of scientific and technological competitiveness to environmental regulation, the level of labor skills has about a 1% positive explanatory power. Compared with column (3), the goodness of fit and overall significance level of column (4) are improved, and the coefficient of determination increases by over one percentage point. That means that in the regression of scientific and technological competitiveness, local fiscal capacity has about 1% of positive impact on variance.

In other words, under given environmental regulations, increasing the productivity of secondary industry and service industry or expanding the relative scale of public fiscal expenditure by local government is conducive to weakening the effect of the “compliance cost” that the investment in pollution abatement makes on scientific advancement. The higher the labor productivity, or the greater the fiscal capacity of local government, the more likely the environmental regulation can realize “innovation compensation”. This shows that, for a resource-based city that wants to protect the natural environment while realizing scientific advancement, it should tap into productivity and stimulate the self-cycling mechanism of human resource accumulation at a detailed level, augment fiscal subsidies for pioneering enterprises shouldering environmental responsibilities, improve the transfer payment between local governments, and strengthen the leading role of the local government in constructing a resource-saving and environmental-friendly society. In addition, compared with the result in Table 2, the coefficient of control variable *Size_it_* is less significant. This is because there is some correlation between the average size of enterprises, labor productivity and fiscal capacity. Other control variables are nearly the same as those in Table 2.

### 4.3. Result of Robust Test

It is worth noting that the intensity of environmental regulation, the level of regional labor skills and regional financial capacity is often endogenous to technological progress. Therefore, the core explanatory variables and regulatory variables contained in the econometric model may be affected by technological changes in the previous period, and the endogenous problems brought about by this may lead to fallacies in the regression results. In order to test the existence of this problem, the Generalized Method of Moments (GMM) is used to re-estimate Equations (2) and (3) to ensure the robustness of the results. The estimation results of GMM are shown in Table 4. *ER_it_*, *lnLP_it_* and *FA_it_* are endogenous explanatory variables, that is, when they are added to the econometric model, the first-order difference of the lag term is taken as the instrumental variable, and the rest of the explanatory variables are regarded as strictly exogenous. The results show that the *p* value of the Arellano-bond AR (2) test indicates that the model is sufficient to avoid residual autocorrelation, and the Sargan test and Hansen test show that there is no over recognition of the instrumental variable. It can be seen that, when the endogenous problem is effectively controlled and there is no over recognition, the effect of *ERit* on *Techit* is basically the same as that in Table 3. Although the absolute value and significance level have decreased, the sign and significance level have not changed. The regulatory effects of *lnLPit* and *FAit* are similar to those in Table 3. All in all, the overall and moderating effects observed in the basic test results are credible.

## 5. Conclusions and Policy Suggestions

The realization of “innovation compensation” for environmental regulations needs the support of a reasonable intensity of regulation and of local economic capacity. Whether resource-based cities in China can protect the natural environment while improving scientific development depends on the non-linear relation between, and regulatory on, the natural environment and scientific development. This paper, based on the empirical data from 33 cities across China from 2008 to 2018, has estimated the non-linear impact that environmental regulations have on scientific competitiveness, and has tested their influence on “innovation compensation”, with labor skills and techniques and local fiscal capacity as regulatory variables.

The results show that: first, a positive U-shaped relation exists between environmental regulation and scientific and technological competitiveness in resource-based cities. That is, when environmental regulation exceeds a certain level, continuously increasing regulatory intensity will obviously enhance scientific competitiveness, but the coefficient of the linear term indicates that the majority of samples are to the left side of the inflection point. Secondly, the higher the non-agricultural labor productivity is, or the greater the fiscal capacity is in a region, the more likely that environmental regulation will take on the nature of “innovation compensation” and the more obvious the effect on promoting scientific competitiveness.

Faced with the pressure of constructing a resource-saving and environmental-friendly society, resource-based cities badly need to strengthen the intensity of regulation regarding industrial pollution and promote technological advancement with the help of “innovation compensation”, thereby driving green, sustainable development with stronger scientific competitiveness.

First, it is necessary to continue to strengthen industrial pollution control and ecological protection investment, and prompt regulatory intensity to cross the inflection point of “U”. For key industries, implementation of strict environmental entry and emission standards is required. In the approval process of new construction projects or renovation and extension projects, total emission control of key pollutants should be taken as an important assessment indicator. Attention must be paid to developing green mines, carrying out strict entry audit and supervision on resource development, and gradually raising the standard of mine construction. As a result, development of ecological civilization in resource-based cities can be promoted to a new height, environmental regulation is enabled to successfully cross the inflection point of “U”, the rigid restrictions on pollution control by enterprises is strengthened, and enterprises become more motivated to realize “innovation compensation”.

Second, focus is needed on stimulating the vigor of production factors, fostering human capital, supporting innovation and entrepreneurship, and comprehensively enhancing labor productivity. Through strengthening governance measures, the earlier viewpoint of treating after polluting needs to be reversed, optimizing urban planning and layout, developing these in an orderly manner, and enhancing the attractiveness of resource-based cities to attract a high-quality workforce. Efforts have been made to establish a human capital enhancement mechanism that stresses introduction of talent, retention of talent and cultivating of talent. Concerted efforts should be made to promote the building of various contingencies related to talent, and to help labor force mobility between industries through the training and introduction of talent. Increased investment in science, education and innovation, giving prominence to the dominant position of enterprises in innovation activities, fully releasing the potential for innovation and stimulating the vitality of factors to promote mass entrepreneurship, with the characteristics of resource-based cities considered, should gradually realize innovation-driven development.

Third, it is necessary to optimize and improve the cross-regional ecological compensation mechanism for resource-rich areas and to strengthen government’s function in environmental regulation. Establishment and improvement of a coordinated evaluation system of resource development and utilization, and urban sustainable development to drive the process of pollution control and ecological restoration in resource-rich areas, are also needed. Local governments should give full play to their functions of guidance, coordination and supervision, and all resource-based cities should arrange and complete the building of a more ecologically aware civilization according to a set plan, giving priority to key points, and ensuring the responsible bodies honor their responsibilities. Regarding areas with a fragile ecology, serious pollution or prominent problems left over from history, it is necessary to strengthen transfer payments among local governments and promote the tilting of resource development benefits to these cities. This will provide growing and regenerative cities with more favorable policies, in order to nurture and develop alternative industries, encourage the establishment of industrial development funds, integrate financial and social funds, and prioritize the approval of livable, ecological and green projects involving local people’s livelihood.

## Figures and Tables

**Table 1:** Description of variables.

Property	Label	Name	Measurement Method	Data Source
Explained variable	*Tech_it_*	Scientific and technological competitiveness	The proportion of the number of employees in scientific research and technical service industries to the total number of employees in i resource-based city in t th year.	China City Statistical Yearbook
Explanatory variable	*ER_it_*	Intensity of environmental regulation	The natural logarithm of the total operating costs of industrial sewage and waste gas treatment equipment of i resource-based city in t th year.	China City Statistical Yearbook
Regulatory variable	*LnLP_it_*	Level of regional labor skills	The natural logarithm of the gross output of secondary and tertiary industries divided by the number of employees of secondary and tertiary industries of i resource-based city in t th year.	China City Statistical Yearbook
	*FA_it_*	Local fiscal capacity	The ratio of public fiscal expenditure to regional GDP of i resource-based city in t th year.	China City Statistical Yearbook
Control variable	*LnGDP_it_*	Level of regional economic development	The natural logarithm of GDP per capita of i resource-based city in t th year.	China City Statistical Yearbook
	*FDI* (Foreign Direct Investment)	Intensity of using foreign investment	The ratio of the amount of actually used foreign investment to the local GDP of i resource-based city in t th year.	China City Statistical Yearbook
	*Size_it_*	The average size of business	Divide the gross value of industrial output above designated size (the main business revenue over 20 million yuan each year) by the number of enterprises and take the natural logarithm for measuring this variable.	China City Statistical Yearbook
	*D_1_* (dummy variable)	Growing city or not?	Yes:1; No: 0.	Planning on Sustainable Development of Resource-based Cities (2013–2020)
	*D_2_* (dummy variable)	Mature city or not?	Yes:1; No: 0.	Planning on Sustainable Development of Resource-based Cities (2013–2020)
	*D_3_* (dummy variable)	Recessionary city or not?	Yes:1; No: 0.	Planning on Sustainable Development of Resource-based Cities (2013–2020)

**Table 2:** Test result of U-shaped relation between the environmental regulation and scientific and technological competitiveness of resource-based cities.

Variable	(1)	(2)	(3)	(4)
*ER*	−0.0225 * (−1.80)	−0.0311 * (−1.86)	−0.0495 * (−1.73)	−0.0872 * (−1.92)
*ER* ^2^			0.6917 ** (2.31)	1.4243 ** (2.14)
*LnGDP_it_*		−0.0030 *** (−2.91)		−0.0030 *** (−2.91)
*FDI*		0.0315 ** (2.41)		0.0324 ** (2.46)
*Size*		0.0005 * (1.91)		0.0004 * (0.83)
*D*_1_		−0.0041 (−0.81)		−0.0042 (−0.82)
*D*_2_		−0.0089 *** (−3.09)		−0.0089 *** (−3.04)
*D*_3_		−0.0088 *** (−2.61)		−0.0088 *** (−2.57)
Constant term	0.0112 *** (9.02)	0.0170 *** (6.39)	0.0113 *** (8.71)	0.0173 *** (6.33)
Hausman test	0.22	2.00	0.17	1.64
Adjusted R^2^	0.0104	0.8657	0.0114	0.8682
Wald value	27.67	52.07	27.77	52.18
Sample size	363	363	363	363

*, ** and ***—significant at the level of 10%, 5% and 1% respectively. In brackets is the statistical magnitude z; the time tendency is controlled in estimation.

**Table 3:** The test result of the regulatory effect between environmental regulations and scientific and technological competitiveness in resource-based cities.

Variable	The Level of Labor Skills and Techniques as Regulatory Variable		The Fiscal Capacity of the Local Government as Regulatory Variable	
	(1)	(2)	(3)	(4)
*ER*	−0.0370 ** (−2.02)	0.1221 (0.44)	−0.0280 * (−1.77)	0.0907 (0.44)
Regulatory variable (*lnLP_it_* and *FA_it_*)	0.0021 * (1.75)	0.0025 * (1.79)	−0.0088 (−1.06)	−0.0049 (−0.46)
Interaction term (*ER_it_* × *lnLP_it_* and *ER* × *FA_it_*)		0.0558 ** (2.18)		0.8293 * (1.95)
LnGDP_it_	−0.0053 *** (−3.17)	−0.0056 *** (−3.18)	−0.0031 *** (−2.97)	−0.0031 *** (−3.02)
*FDI*	0.0267 ** (2.00)	0.0271 ** (2.03)	0.0331 ** (2.52)	0.0332 ** (2.52)
*Size*	0.0004 (0.90)	0.0004 (0.83)	0.0005 (1.05)	0.0006 (1.10)
*D*_1_	−0.0046 (−0.89)	−0.0047 (−0.91)	−0.0036 (−0.71)	−0.0037 (−0.72)
*D*_2_	−0.0092 *** (−3.15)	−0.0094 *** (−3.16)	−0.0086 *** (−2.96)	−0.0087 *** (−2.91)
*D*_3_	−0.0091 *** (−2.65)	−0.0092 *** (−2.66)	−0.0083 ** (−2.46)	−0.0084 ** (−2.42)
Constant term	0.0127 *** (3.45)	0.0117 *** (2.92)	0.0174 *** (6.47)	0.0168 *** (5.84)
Hausman test	1.51	1.56	3.65	2.97
Adjusted R^2^	0.8695	0.8791	0.8546	0.8659
Wald value	55.13	55.28	53.22	53.24
Sample size	363	363	363	363

*, ** and *** means significant at the level of 10%, 5% and 1% respectively. In the bracket is the statistical magnitude z; the time tendency is controlled in estimation.

**Table 4:** Robustness test results based on System Generalized Method of Moments GMM.

Variable	The Level of Labor Skills and Techniques as Regulatory Variable		The Fiscal Capacity of the Local Government as Regulatory Variable	
	(1)	(2)	(3)	(14)
*ER*	−0.0293 ** (−2.02)	0.1220 (0.40)	−0.0279 * (−1.76)	0.0910 (0.48)
Regulatory variable (*lnLP_it_* and *FA_it_*)	0.0020 * (1.69)	0.0031 * (1.79)	−0.0077 (−1.10)	−0.0050 (−0.45)
Interaction term (*ER_it_ × lnLPit* and *ER × FA_it_*)		0.0561 ** (2.21)		0.8310 * (1.96)
*LnGDP_it_*	−0.0051 *** (−3.21)	−0.0056 *** (−3.18)	−0.0029 *** (−2.89)	−0.0033 *** (−3.00)
*FDI*	0.0271 ** (2.01)	0.0269 ** (2.02)	0.0330 ** (2.52)	0.0334 ** (2.52)
*Size*	0.0004 (0.91)	0.0005 (0.82)	0.0005 (1.10)	0.0006 (1.09)
*D_1_*	−0.0046 (−0.89)	−0.0046 (−0.91)	−0.0033 (−0.71)	−0.0038 (−0.72)
*D_2_*	−0.0089 *** (−3.23)	−0.0095 *** (−3.21)	−0.0086 *** (−2.87)	−0.0087 *** (−2.90)
*D_3_*	−0.0087 *** (−2.73)	−0.0090 *** (−2.74)	−0.0079 ** (−2.55)	−0.0078 ** (−2.51)
*AR(2)*	0.6540	0.0890	0.5560	0.0860
Sargan test	0.0990	0.8900	0.1860	0.1220
Hansen test	0.4690	0.1380	0.1400	0.2260
Sample size	363	363	363	363

Z statistic is shown in brackets; two-step method is used in estimation; constant term and first-order lag term of explained variable are included in estimation, and the estimation results are not listed; regional and annual variables are controlled in estimation; *p*-value is shown in each test value; *, **, and *** represent significant at the 10%, 5%, and 1% levels respectively.

## Appendix A

**Table A1:** Names and Types of 33 Sample Cities.

NO.	Cities	Types
1	Tang Shan	regenerative city
2	Han Dan	mature city
3	Da tong	mature city
4	Yang Quan	mature city
5	Chang Zhi	mature city
6	Lin Fen	mature city
7	Bao Tou	regenerative city
8	Chi Feng	mature city
9	An Shan	regenerative city
10	Fu Shun	recessionary city
11	Ben Xi	mature city
12	JI Lin	mature city
13	Mu Danjiang	mature city
14	Xu Zhou	regenerative city
15	Hu Zhou	Mature city
16	Ma Anshan	regenerative city
17	Zi Bo	regenerative city
18	Zao Zhuang	recessionary city
19	Ji Ning	mature city
20	Tai Shan	mature city
21	Luo Yang	regenerative city
22	Ping Dingshan	mature city
23	Jiao Zuo	recessionary city
24	Shao Guan	recessionary city
25	Pan Zhihua	mature city
26	Lu Zhou	recessionary city
27	Qu Jing	mature city
28	Tong Chuan	recessionary city
29	Bao Ji	mature city
30	Xian Yang	growing city
31	Yan An	growing city
32	Shi Zuishan	recessionary city
33	Ke Lamayi	mature city
